# Not All Math Activities Are Equal in Terms of Gender Stereotypes

**DOI:** 10.1007/s11199-026-01645-2

**Published:** 2026-03-18

**Authors:** Megan Merrick, Andrew D. White, Amanda B. Diekman, Emily R. Fyfe

**Affiliations:** 1 Department of Psychological and Brain Sciences, Indiana University, Bloomington, IN US; 2 Department of Counseling Psychology, Social Psychology, and Counseling, Ball State University, Muncie, IN US

**Keywords:** Mathematics, Gender stereotypes, Numeracy, Spatial reasoning, Patterning

## Abstract

The “math as male” stereotype is societally pervasive and emerges early in development. However, math is a broad and multifaceted domain that requires proficiency in several different cognitive skills. The current study explored the gender stereotypes associated with toys linked to the cognitive skills of numeracy, spatial reasoning, and patterning. Across three studies (*N* = 878), adults viewed toys associated with each cognitive skill and reported who would prefer them: boys, girls, U.S. parents buying for boys versus girls, and self as a child. The gendering of toys varied by toy. The most consistent alignment was between spatial reasoning toys and boys. Results for numeracy and patterning toys were more mixed, but sometimes neutral or female-leaning. There also tended to be a shift when reporting about others’ preferences versus own preferences, with a weaker male stereotype for the latter. Understanding how adults associate these cognitive skills with gender has important implications for research in mathematics education and belonging within STEM fields.

Despite increasing awareness of the importance of math skills for all students, gender disparities in math attitudes and interest persist. In children as young as six-years-old, girls estimate lower math abilities and report more negative attitudes towards math than boys despite gender similarities in math performance (Fredricks & Eccles, 2002; Jacobs et al., 2002; Muzzatti & Agnoli, 2007). From children to adults, people tend to associate the concept of “math” more strongly with men and boys (e.g., Cvencek et al., 2011; Nosek et al., 2002). However, the strength of the “math as male” stereotype can vary. For example, gender stereotypes about male *interest* in quantitatively focused fields are more powerful than stereotypes about male *ability* (Master et al., 2021). Further, gender stereotypes vary across STEM fields with greater gender disparities in fields where stronger masculine defaults exist (Cheryan et al., 2017, 2025). In the current study, we ask whether gender stereotypes vary when the construct of “math” is unpacked.

Early math researchers recognize that mathematics knowledge is a multi-faceted construct that requires specific skills, including numeracy, spatial reasoning, and patterning (Di Lonardo Burr et al., 2022; LeFevre et al., 2010). The goal of the current study is to ask if all math activities are “equal” in terms of gender stereotypes by investigating gendered beliefs about toys associated with these three cognitive skills. To do so, we ask adult participants about their beliefs about who would enjoy childhood toys associated with each of these skills. Assessing beliefs about toys represents an important and ecologically valid measure as adults select toys for children that in turn structure their learning environments.

## Gender Stereotypes of Mathematics

Highly mathematics-intensive fields such as math, computing, and engineering continue to be male-dominated at the undergraduate, graduate, and workforce levels (National Science Board, 2022). A great deal of past literature suggests that these disparities have emerged partly due to pervasive stereotypes of mathematics as a male-stereotypic domain (Cheryan, 2012; Cheryan et al., 2017; Steffens et al., 2010). Associating math with men and boys more than with women and girls has several negative effects for women and girls’ engagement with math, and these effects emerge even among children ages 6–10 (Cvencek et al., 2011; Nosek et al., 2002). Gender stereotypic beliefs of “math as male” can also be perpetuated by parents and teachers, as their interactions with children in early math environments shape children’s math attitudes and ability beliefs (Bhanot & Jovanovic, 2005; Starr et al., 2022). For instance, when their children succeed in math, parents often attribute this success to effort for daughters but to talent and innate ability for sons (Gladstone et al, 2018; Räty et al, 2002; Yee & Eccles, 1988).

However, mathematics is a broad domain that encompasses competence in a variety of cognitive skills. If gender stereotypes are more weakly held about some of those cognitive skills, this variability may provide a useful leverage point for more gender-equitable engagement. We investigate the gendered perceptions of specific cognitive skills that are often associated with mathematics–namely, numeracy, spatial reasoning, and patterning (e.g., Di Lonardo Burr et al., 2022; Rittle-Johnson et al., 2019).

## Numeracy

Numeracy is knowledge of whole numbers and how numbers relate to each other. Numeracy includes activities such as counting, comparing magnitudes, and operations (Purpura & Lonigan, 2013). An extensive literature provides evidence that early numeracy is a predictor of later math achievement (Jordan et al., 2007, 2009, 2010). The literature generally suggests that there are not strong differences between boys and girls in their numeracy knowledge. For example, one study on a wide variety of numeracy tasks (e.g., counting, number comparison, calculations) with over 1300 boys and girls aged 6–13 found little evidence for gender differences; and Bayesian analyses generally supported the conclusion that gender differences did not exist (Hutchison et al., 2019).

Despite these data, there are several reasons to expect a gender stereotype to emerge for numeracy. First, many adults define numeracy similarly to how they define the broader term of mathematics (Merrick et al., 2026). Second, measures of math stereotypes that demonstrate a “math as male” bias often equate math with numeracy. For example, when assessing children’s associations of math with boys versus girls, the “math” words are often related to numeracy (e.g., addition, numbers; Cvencek et al., 2011). Thus, just as math is stereotyped as male, we hypothesize that numeracy will also be stereotyped as male.

## Spatial Reasoning

Spatial reasoning encompasses a variety of skills including the ability to visualize, understand, and manipulate objects (Uttal et al., 2013), and these skills can vary on several dimensions (e.g., intrinsic-extrinsic, static-dynamic; Newcombe & Shipley, 2014). Spatial reasoning is considered a core predictor of mathematics knowledge as many mathematics tasks require spatial representations like shapes, angles, and measurement (Mix & Cheng, 2012). There is some reason to anticipate gender stereotypes about spatial reasoning. For example, tasks that involve mental rotation of objects (an intrinsic-dynamic task) are among the larger cognitive gender differences favoring boys (Hyde, 2005; Voyer et al., 1995). In fact, in a meta-synthesis of gender differences across the psychological literature, mental rotation ability was the second largest gender difference (favoring males) detected across the literature (Zell et al., 2015). This gender difference appears to be early developing, as one study reported a small male advantage in mental rotation performance during childhood that subsequently increased with age, reaching a moderate effect size during adolescence (Lauer et al., 2019). However, a male advantage in spatial skills is not always evident. For example, in a mental folding task (also considered an intrinsic-dynamic task, but requires non-ridge transformations), the male advantage disappears (Harris et al., 2013).

These reported differences in spatial reasoning favoring boys have been partially explained by boys’ greater experience with spatially-oriented toys, such as video games or throwing and catching objects (an extrinsic-dynamic task; Terlecki & Newcombe, 2005; Uttal et al, 2013). To the extent that observers see boys more than girls engaging in spatially-oriented activities and demonstrating spatial skill, they may hold beliefs that boys have greater spatial reasoning than girls. To date, some research finds evidence of gender stereotypes about spatial reasoning. For example, a study of 10–12-year-old children’s beliefs about spatial abilities found that on explicit measures, both boys and girls associated spatial ability with boys, whereas on implicit measures, boys associated spatial ability with boys whereas girls showed no gendered associations (Vander Heyden et al., 2016). Adults also report gender stereotypes about spatial skills. For example, adult men held stronger beliefs linking spatial skills to men (Van der Ham & Koutzmpi, 2023), and parents attributed greater spatial ability to sons than daughters (Muenks et al., 2020). Given the current state of evidence, we hypothesize that spatial reasoning will also be stereotyped as male.

## Patterning Skills

Patterning skills include identifying and extending predictable sequences, often that repeat (AB-AB-AB) or increase systematically (AB-ABB-ABBB; Rittle-Johnson et al., 2013). Patterning is a core skill in mathematics because it requires the ability to identify the underlying structure of a task (e.g., Mulligan & Mitchelmore, 2009). Empirical work shows that patterning skills are associated with mathematics achievement both concurrently (e.g., Fyfe et al, 2017; Wijns et al., 2019) and longitudinally (e.g., Nguyen et al., 2016; Rittle-Johnson et al., 2017).

In terms of gendered differences in patterning skills, previous literature is sparse. Many studies on children’s patterning skills do not examine or report the effect of gender on performance. At least one study reported a null effect (Güven et al., 2021) and one study reported a male advantage in first grade children, but the patterns required knowledge of the count sequence and the alphabet (Patterson et al., 2015). More recently, three studies that examined patterning in children ages 4 to 6 reported female advantages for creating or solving repeating pattern tasks (De Keyser et al., 2020; Schreiber, 2025; Wijns et al., 2019). This limited evidence suggests a lack of consensus about gendered gaps in patterning skill, but potentially a trend favoring girls.

To our knowledge, no research has directly examined gendered beliefs about patterning, but there may be reasons to expect gender stereotypes to be attenuated or even reversed. For example, observers’ own experiences may parallel the results in the literature about actual gender differences in patterning with a slight female advantage. Also, patterning is a visible component in arts and crafts activities, which are frequently engaged by girls and women. Indeed, undergraduate students identified various skills within the textile arts (e.g., embroidery, crochet, knitting, quilting) as female-stereotypic (Bennett-Pierre & Gunderson, 2023; Newcombe et al., 1983). Thus, we hypothesize that gender stereotypes about patterning skills will be attenuated or potentially reversed, resulting in a “patterning as female” stereotype.

## Toys as a Socialization Space

Toys play an important role in structuring children’s early learning environment. Children’s interaction with toys can set the stage for the development of important cognitive skills. For example, children who more frequently played with puzzles and blocks showed greater spatial ability (Jirout & Newcombe, 2015). Even playing a linear number board game for a total of one hour led to noticeable improvements in numerical knowledge in preschoolers (Ramani & Siegler, 2008). It is not just play alone that benefits children’s cognitive skills; rather, the specific features of toys may matter. For example, toys that involve building may support spatial reasoning while toys that involve counting spaces on a game board may support numeracy. Given this, it is important to consider which children have access to different toys because different toys may foster distinct cognitive skills during their childhood.

Toys can also convey powerful gender norms about what is “appropriate” for girls and boys. For example, adults’ decisions about what toys their children play with can vary by child gender. Parents rated cross-gender toys as least desirable when compared to same-gender or gender-neutral toys; and this pattern was exacerbated for parents who endorsed greater gender normative attitudes (Kollmayer et al., 2018). Exploring perceptions of children’s toys can thus inform the psychology underlying how adults structure children’s early learning environments.

## Current Study

The goal of the current study is to examine adults’ views of three key cognitive skills related to mathematics–numeracy, spatial reasoning, and patterning–and their gendered expectations of each skill. Across three studies, we presented adults with educational toys that highlighted one of the specific cognitive skills. In Study 1, college students rated how much a boy or girl would like the toys, which were selected for their association with numeracy, spatial reasoning, or patterning. In Study 2, participants were shown different puzzle toys, each randomly assigned to be framed as supporting numeracy, spatial reasoning, or patterning, and asked to rate how likely a parent in the United States would be to buy each puzzle toy for their son or daughter. In Study 3, participants were shown a neutral toy, which was randomly assigned to be framed as supporting numeracy, spatial reasoning, or patterning, and asked to rate the likelihood of parents buying them for their son or daughter. The assigned framing of toys across Studies 2 and 3 allowed us to isolate the influence of the label of the cognitive skill.

We developed three hypotheses which spanned across all three studies: Adults will perceive numeracy (H1) and spatial reasoning (H2) as male-stereotypic domains and report higher preferences for numeracy and spatial toys for boys relative to girls. Also, the male-stereotypic association will be attenuated or reversed for patterning (H3) and adults will potentially report a higher preference for patterning toys for girls relative to boys.

## Study 1

## Method

We conducted an initial study with the use of educational toys to explore the gendered perceptions of each cognitive skill. The educational toys were selected by the research team from the “Lakeshore Learning” website, a popular retailer with educational products for both teachers and parents. We selected four toys for each target skill where these terms were prevalent in the toy’s description.

### Participants

Original participants included 177 students with consent from a Midwestern university in the United States who were part of the participant pool (SONA). Nine participants were removed from the sample for taking less than five minutes to complete the survey. The final analytic sample contained 168 participants. A sensitivity analysis using G*Power 3.1.9.7 (Faul et al., 2007) indicated that our sample could detect an effect size of *f* = .08, a small effect, for a 2 x 3 repeated measures ANOVA (α = .05, power = .80).

Within this sample, 115 (68.5%) of the participants identified as women, 48 (28.6%) as men, and five identified as nonbinary or transgender. The sample identified as White (123, 73.2%), Asian (14, 8.3%), Black (10, 6.0%), Latinx (9, 5.4%), Biracial/Multiracial (8, 5.4%), Middle Eastern (2, 1.2%), and Native American (1, 0.6%). Self-reported socioeconomic status averaged 6.39 on a scale ranging from 1 (*Lowest status*) to 10 (*Highest status*; *SD* = 1.55). The median sample age was 19 years old, with a range from 17 to 24 years old.

### Procedure and Materials

Participants completed an online survey which took approximately 15 minutes. They were told they would see “a variety of children’s education games” and be asked who would enjoy these games the most. Participants were then shown 12 educational toys in a randomized order. Based on the toy descriptions on the website, we selected four toys to represent numeracy, four toys to represent spatial skills, and four toys to represent patterning. See the Appendix for pictures of the toys. For each toy, participants first rated how much the toy promoted early learning in four areas (i.e., math skills, literacy skills, problem solving, critical thinking) using a five-point Likert scale. Then, they answered three primary questions about toy “liking” using a Likert scale from 1 (*Not at all*) to 5 (*A lot*). The first question was about their own preferences: “How much would you have liked this toy as a child?” They also explained their response to this question in an open-ended text box: “What about this toy would you have liked or not liked when you were a child?” The last two “liking” questions were their beliefs about other children: “How much would a boy (girl) like this toy?”

At the end of the survey participants answered key demographic questions and were asked to leave any comments or issues they had about the survey if desired. The full list of survey items is available on the Open Science Framework (OSF) at https://osf.io/zrjm4.

## Results

### Perceptions of Children’s Toy Liking

**Fig. 1:**
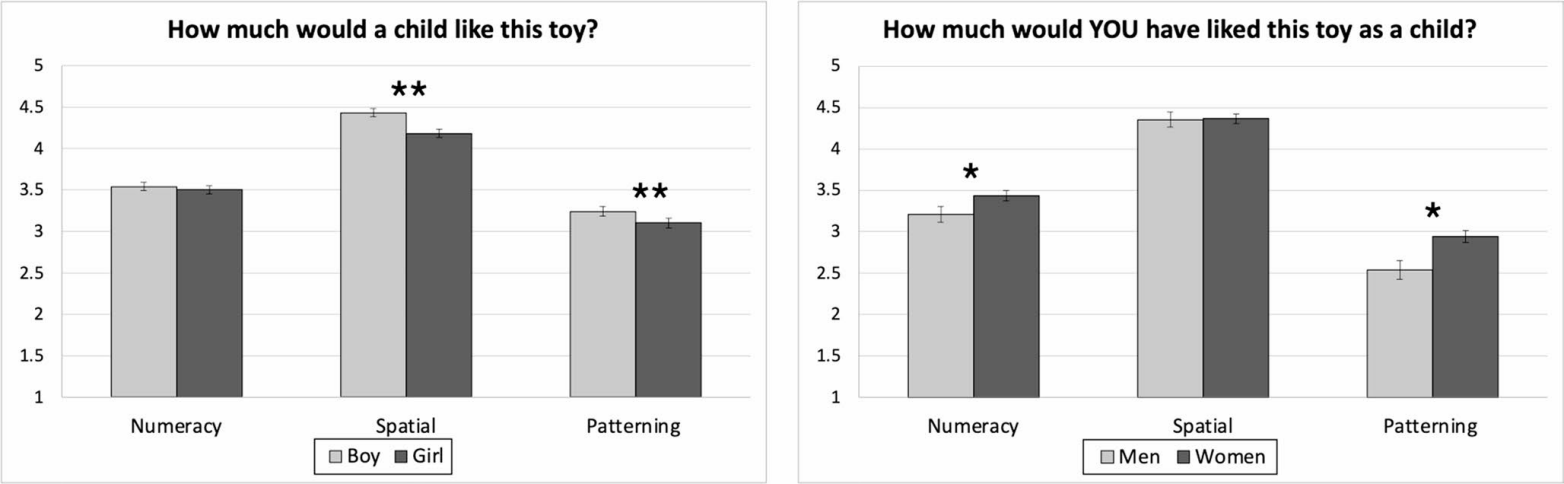
Panel **A** - Perceptions of how much a child (boy or girl) would like the toy as a function of cognitive skill*.* Panel **B** - Ratings of how much the participant would like numeracy, spatial, and patterning toys as a child. *Note.* Error bars represent ± 1 standard error. * indicates *p *< .05, ** indicates *p *< .001. The sample consisted of *n *= 163 in Panel **B**

First, we examined beliefs about children’s toy liking across gender and skill. We conducted a 2 x 3 repeated measures ANOVA with target gender (boy, girl) in the item wording, and target skill (numeracy, spatial, patterning) in the toys presented, as within-subject factors. We averaged ratings of liking across the four toys within each skill. As depicted in Fig. 1, there was a main effect of gender, *F*(1, 167) = 63.94, *p* < .001, η_p_^2^ = .28, a main effect of skill, *F*(2, 167) = 223.51, *p* < .001, η_p_^2^ = .57, and a two-way interaction, *F*(2, 167) = 16.75, *p* < .001, η_p_^2^ = .09.

Post-hoc tests revealed significant differences for spatial toys and patterning toys, but not for numeracy toys. Adults reported that boys would like spatial toys more than girls, *t*(167) = 9.04, *p* < .001, *d* = 0.70, and this was true for each of the four spatial toys, *p*s <.05. Adults also reported that boys would like patterning toys more than girls, *t*(167) = 4.70, *p* < .001, *d* = 0.36. However, this gender gap for patterning was driven by a single toy, and the other three pattern toys showed a reverse trend. The patterning toy that featured “nuts and bolts” had higher ratings for boys (*M* = 3.82, *SD* = 1.05) than for girls (*M* = 2.83, *SD* = 0.92), *t*(167) = 12.07, *p* < .001, *d* = 0.93. However, adults reported higher liking ratings for girls than boys for the patterning toys that featured beading (for girls *M* = 3.32, *SD* = 1.00; for boys *M* = 3.00, *SD* = 0.97), *t*(167) = 5.39, *p* < .001, *d* = 0.42, and which featured fruits and an animal (for girls *M* = 3.11, *SD* = 1.01; for boys *M* = 3.00, *SD* = 0.96), *t*(167) = 2.84 *p* < .001, *d* = 0.22. There was not a difference in liking for patterning toys which featured shape matching (for girls *M* = 3.16, *SD* = 1.02; for boys, *M* = 3.13, *SD* = 0.98), *t*(167) = 1.00, *p* = .319, *d* = 0.08. There was no overall target gender effect for numeracy toys, *t*(167) = 1.61, *p* = .109, *d* = 0.12, and this was true for three of the four numeracy toys, *p*s > .05. The exception was a higher rating for boys than girls on the toy featuring play-dough, *p* < .001.

### Participants’ Retrospective Beliefs About Own Toy Liking as Children

Next, we examined adults’ liking for toys when considering thinking about themselves as children. Five participants were excluded from this analysis because they identified as nonbinary or transgender (analytic *n* = 163). We conducted a 2 x 3 mixed ANOVA with participant gender (men, women) as a between-subject factor and skill (numeracy, spatial, patterning) as a within-subject factor. There was a main effect of participant gender, *F*(1, 161) = 6.04, *p* = .015, η_p_^2^ = .36, a main effect of skill, *F*(2, 161) = 275.04, *p* < .001, η_p_^2^ = .63, and an interaction between the two, *F*(2, 161) = 3.88, *p* < .022, η_p_^2^ = .02. See Fig. 1. Post-hoc tests revealed significant differences in liking for numeracy toys and patterning toys, but unexpectedly not for spatial toys. Women reported liking both patterning toys, *t*(161) = 3.01, *p* = .003, *d* = 0.52, and numeracy toys, *t*(161) = 1.98, *p* = .049, *d* = 0.34, more than men did.

### Open-Ended Responses

To shed light on adults’ ratings, we conducted descriptive analyses on their open-ended responses in which they described “what about the toy” they would have liked or not liked when they were a child. We created a rubric to categorize the features they mentioned most frequently. Using a data-driven approach, we identified five primary features that contributed to their liking of the toy. These included (1) general perceptual features like color or shape, (2) specific toy components like beads, blocks, or dough, (3) physical interaction, (4) educational components like supporting learning or creativity, and (5) the topic concept like number or patterns. A full description and rubric along with all descriptive results are provided in the supplemental file on OSF at https://osf.io/zrjm4.

We noticed several key themes among participants reports of what they liked or did not like about the toys. First, the responses for the four spatial toys were all very similar to each other, meaning that for each of the spatial toys, the most common feature to identify was the physical interaction (e.g., building, putting together), and it was highly prevalent (mentioned in > 50% of responses for each toy). Also, it was more common to mention the learning features of the spatial toys (25% of responses) relative to the numeracy (12.5%) or pattern toys (8.25%). Second, the numeracy toys were also perceived as similar to each other in that the first or second most common feature for each toy was the numeracy topic (e.g., liking the number or math component) and it was somewhat prevalent (e.g., mentioned in > 20% of responses for each toy). Third, the patterning toys were perceived as more distinct from each other - for some pattern toys it was common to name the general perceptual features (e.g., bright colors) and for others it was common to focus on the specific toy parts (e.g., the tools, the koala). Further, across the four pattern toys, the prevalence rates for mentioning these five features were low, meaning these responses tended to be vague (e.g., doesn’t look that fun, not sure) and not focused on these features.

### Perceptions of Supporting Math

Finally, we examined adults’ ratings of how much the toy promoted four areas of early learning (math skills, literacy skills, problem solving, critical thinking). Because the main goal of our investigation is to explore the gendered stereotypes of cognitive skills related to math, here we focus our analysis on adults’ ratings of how much the toy promoted early math skills specifically. Analyses for the other three areas of early learning (literacy skills, problem solving, critical thinking) can be found in the supplemental materials.

To examine adults’ ratings of how much the toy promoted early math skills, we conducted a 2 x 3 mixed ANOVA with participant gender (women, men) as a between-subject factor and skill (numeracy, spatial, patterning) as a within-subject factor. There was no main effect of participant gender, *F*(1, 161) = 3.84, *p* = .052, η_p_^2^ = .02. There was a main effect of skill, *F*(2, 161) = 322.29, *p* < .001, η_p_^2^ = .67, but no interaction between participant gender and skill, *F*(2,161) = 0.30, *p* = .701, η_p_^2^ = .00. Post-hoc tests revealed higher ratings for numeracy toys promoting early math skills (*M* = 4.41) relative to spatial toys, *M* = 2.46; *t*(167) = 21.80, *p* < .001, and patterning toys, *M* = 2.53; *t*(167) = 22.49, *p* < .001. This is consistent with people aligning numeracy with math.

## Discussion

Our initial study revealed several key findings. First, when considering boys and girls, adults hold the “spatial as male” stereotype, reporting that boys would prefer spatial toys more than girls. However, this gender difference was not apparent when rating their own liking of the spatial toys. Second, there was a trend for adults to hold beliefs that reversed from traditional gender expectations about math when they considered patterning. Women reported liking the pattern toys more than men, and on two of the patterning toys the adults reported that girls would like them better than boys. Third, the results for numeracy were unexpected, as adults did not report boys liking the numeracy toys better than girls, and women reported marginally liking them better as children themselves. Together, these results suggest that stereotypes differ by skill, but they may also differ when thinking about others versus oneself. Further, the specific toy features clearly mattered (e.g., the color or shape, whether it has tools or beads). Spatial toys were consistently identified by their high degree of physical interaction, relative to numeracy and patterning toys.

## Study 2

Following Study 1, we conducted Study 2 with a larger sample size and a similar within-subject design but with different toys and with explicit labels. Specifically, the manipulation consisted of framing a toy as supporting numeracy, spatial reasoning, or patterning. We also introduced a control condition where the toy was framed to be “just for fun.” We wanted stimuli that (a) seemed plausible for each verbally framed label attached to it, and (b) were more spatial in nature given that one of the strongest findings from the pilot study was the “spatial as male” association. We reasoned that demonstrating a verbal label (e.g., numeracy, patterning) influences perceptions even when applied to a spatial-leaning toy would be strong evidence of the power of those labels.

We selected four “puzzle” toys that satisfied both criteria. For example, the puzzle toys had pieces that could be fit together or stacked (spatial), counted (numeracy), or sorted or arranged in sequences (patterns). Also, these types of puzzle toys are often considered “spatial toys” and playing with them is related to children’s spatial skills (e.g., Levine et al., 2012). Further, all four of the spatial toys in the pilot study were larger toys that required building, letting us explore whether the “spatial as male” bias existed for other spatial-leaning toys that afforded different actions.

## Method

### Participants

Original participants included 444 students with consent from a Midwestern university in the United States. The participants were recruited using the university participant pool (SONA). Thirteen participants were removed from the sample for taking less than five minutes to complete the survey. The final analytic sample contained 431 participants. A sensitivity analysis using G*Power 3.1.9.7 (Faul et al., 2007) indicated that our sample could detect an effect size of *f* = .045, a small effect, for a 2 x 4 repeated measures ANOVA (α = .05, power = .80).

Within this sample, 322 (74.7%) of the participants identified as women, 96 (22.3%) as men, and 13 (3.0%) identified as nonbinary or transgender. The sample identified as White (300, 69.6%), Asian (58, 13.5%), Biracial/Multiracial (32, 7.4%), Latinx (18, 4.2%), Black (16, 3.7%), and Middle Eastern (4, 0.9%). Three participants (0.7%) choose to not report their race. Self-reported socioeconomic status (SES) averaged 6.40 on a scale ranging from 1 to 10 (*SD* = 1.62). The median sample age was 19 years old, with a range from 17 to 31 years old.

### Procedure and Materials

The four toy stimuli were selected from products available on Amazon and are presented in the Appendix. We selected stimuli that (a) seemed plausible for each verbal frame, and (b) were more spatial in nature to provide a rigorous test of the labels. The four “puzzle” toys selected from Amazon.com satisfied both of these desires. The types of puzzle toys we selected all had pieces that could be arranged, fit together, or stacked (i.e., making them appropriate for the spatial label). However, the pieces could also be counted or measured (i.e., making them appropriate for the numeracy label), or could be sorted or arranged in sequences by color or shape (i.e., making them appropriate for the patterning label). In addition to ensuring that any of the verbal frames were plausible, we selected puzzle toys given that they tend to be inherently spatial in nature - they are often considered “spatial toys” and playing with them is related to children’s spatial skills (e.g., Levine et al., 2012). We reasoned that if a verbal label - like numeracy or patterning - influenced perceptions even when applied to a spatial-leaning toy, then it was a strong testament to the power of those labels with regard to gender perceptions.

Participants completed an online survey which took approximately 15 minutes to complete. First, they were told they would see various educational toys and to think about which children would be interested in those toys. They then completed four blocks of tasks. Within each block, the tasks were the same but focused on one of four constructs: numeracy, spatial reasoning, patterning, or “just for fun.” At the beginning of the block, they were introduced to one of the key labels and a definition was provided. Then participants were asked to generate a list of three to five activities, toys, or games that might support this construct in children. Next, participants were presented with a picture of one of the four puzzle toys and were told that this toy was designed to improve children’s early learning of that construct.

Participants answered three primary questions about the toy. The first two questions were about the likelihood that parents would buy that toy on a scale from 1 (*Extremely unlikely*) to 5 (*Extremely likely*): “In general, how likely are parents in the US to buy this toy for their son (daughter)?” The third question was about their own preferences: “How much would you have liked this toy as a child?” from 1 (*None at all*) to 5 (*A great deal*). Participants also answered five additional items which we do not report on here. Three of the unreported items used a Likert scale to determine how much participants believed that each toy promoted early learning in three areas (math achievement, literacy achievement, general academic achievement). Two other unreported items were free response items, asking participants to first “describe how you might play with this toy with a Kindergartener,” and then “describe the kind of child who would enjoy this toy and why they might enjoy it.”

Participants then repeated these tasks in the remaining three blocks, but about the other constructs. The order of constructs was randomized across participants. The pairing of a specific construct with a specific toy was also randomized across participants. For example, for participant A, the first puzzle toy may have been labeled patterning but for participant B that same puzzle toy may have been labeled just for fun. Both the puzzle toys and the skills were randomized where each participant saw each of the four toys in a random order matched with a specific skill, also presented in a random order.

At the end of the survey participants answered key demographic questions and were asked to leave any comments or issues they had about the survey if desired. The full list of survey items is available on the Open Science Framework (OSF) at https://osf.io/zrjm4.

## Results

### Perceptions of Parents’ Willingness to Buy Toy

**Fig. 2:**
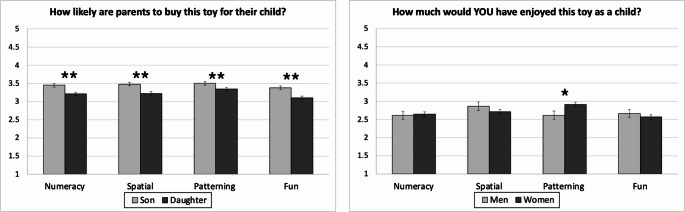
Panel **A** - Perceptions of parents’ (in the US) willingness buy the puzzle toys for child (boy or girl), as a function of cognitive skill*. *Panel **B** - Ratings of how much the participant would have enjoyed the toy as a child, as a function of cognitive skill. Note*.* Error bars represent ± 1 standard error. * indicates *p *< .05, ** indicates *p *< .001. The sample consisted of *n *= 418 in Panel **B**

To examine differences in ratings across gender and skill, we conducted a 2 x 4 repeated measures ANOVA with target gender (boy, girl) in the item wording, and target skill framing (numeracy, spatial, patterning, fun) in the toys presented, as within-subject factors. There was a main effect of target gender, *F*(1, 425) = 90.42, *p* < .001, η_p_^2^ = .18, and a main effect of skill framing, *F*(3, 425) = 3.75, *p* = .011, η_p_^2^ = .01. However, the interaction between the two was not significant, *F*(3, 425) = 2.05, *p* = .105, η_p_^2^ = .01. As shown in Fig. 2, the effect of skill framing was driven by less willingness to buy the toy for toys labeled as “fun.” The main effect of gender was due to participants reporting that parents would be more likely to buy these puzzle toys for their sons than for their daughters. Indeed, pairwise comparisons showed that adults believed that parents would likely buy toys for sons more than daughters within each skill framing condition, *p*s < .001.

### Rating of Their Own Toy Enjoyment as Children

Next, we examined adults’ liking for the toys when imagining themselves as children. Thirteen participants were excluded from these analyses because they identified as nonbinary or transgender (analytic *n* = 418). We conducted a 2 x 4 mixed ANOVA with participant gender (women, men) as a between-subject factor and toy skill framing (numeracy, spatial, patterning, fun) as a repeated within-subject factor. There was not a significant main effect of participant gender, *F*(1, 407) = 0.05, *p* = .827, η_p_^2^ = .90 nor a significant main effect of skill framing, *F*(3, 407) = 2.28, *p* = .083, η_p_^2^ = .01. However, there was a significant interaction between the two, *F*(3, 407) = 2.81, *p* = .043, η_p_^2^ = .01, as displayed in Fig. 2. Unpacking this interaction, gender differences only emerged for ratings of patterning toys, *t*(412) = −2.19, *d* = −0.26, *p* = .029, in which women reported greater imagined enjoyment as a child than men.

## Discussion

Study 2 replicated several key findings of Study 1. First, we believe the results are consistent with the "spatial as male" stereotype found in Study 1. For toys labeled as supporting spatial reasoning, participants reported that parents would be more likely to buy these puzzle toys for their sons than for their daughters. Again, however, this gender stereotype was not apparent when adults rated their own liking of the spatial toys. Second, as in Study 1, the specific toy features seemed to matter. Recall that all the toys in Study 2 were some form of puzzle and inherently spatial in nature, and we found that adults thought parents would be more likely to buy all four toys for their sons than for their daughters, regardless of how they were labeled. To be clear, the labels may still matter somewhat in addition to the toy features. For example, the toys labeled as "just for fun" were rated as least likely to be purchased relative to the toys labeled as supporting numeracy, spatial reasoning, or patterning, even though the toys were identical. Fourth, like in Study 1, adults held a reverse stereotype for patterning: Contrary to traditional stereotypes about math, women reported liking the toys labeled as patterning slightly more than men.

## Study 3

In Study 3, we wanted to isolate the effect of the labels of the toys and remove any variation due to toy features. Thus, to increase experimental control, all participants viewed the same toys and we manipulated the toy labels in a between-subject design. We selected a set of toy bears that were neutral in appearance and not inherently linked to any of the math skills. The experimental manipulation consisted of framing these toy bears as supporting numeracy, spatial reasoning, patterning skills, or “just for fun.” The image of the toy bears was designed by the research team, and purposefully displayed in configurations to afford activities related to each construct. For example, the image showed that the bears could easily be stacked (spatial), counted (numeracy), alternated in color (patterns), or played with (fun). An added strength to Study 3 is that the sample was collected via Prolific so that the participants were more representative of the U.S. population, in terms of age and race, relative to the Study 2 college sample.

## Method

### Participants

Original participants included 279 adults from the United States collected via Prolific. They were randomly assigned to a numeracy condition (*n* = 64), a spatial condition (*n* = 73), a patterning condition (*n* = 70), or a fun control condition (*n* = 72). An a priori power analysis using G*Power 3.1.9.7 (Faul et al., 2007) indicated that we needed a sample size of 280 to detect a small effect (*f* = .10, α = .05, power = .80) for a 2 x 4 mixed-measures ANOVA.

Within this sample, 178 (63.8%) of the participants identified as women, 90 (32.3%) as men, and 11 (3.9%) identified as nonbinary or transgender. The sample identified as White (171, 61.3%), Asian (43, 15.4%), Black (27, 9.7%), Biracial/Multiracial (21, 7.5%), Latinx (15, 5.4%), Middle Eastern (1, 0.4%), and Native American (1, 0.4%). The sample’s self-reported SES averaged 5.22 on a scale ranging from 1 to 10 (*SD* = 1.67). The median sample age was 31 years old, with a range from 18 to 70 years old.

### Procedure and Materials

Participants completed an online survey which took approximately five minutes to complete. They were told they would see an educational toy and to think about which children would be interested in it. All participants were shown the exact same image of the toy bears (see the Appendix), but it was described differently depending on their condition.

Participants were introduced to one of three key academic terms (numeracy, spatial reasoning, patterning skills) or a control term (fun) and a corresponding definition. They were then shown a picture of the toy bears along with descriptions of how a child might play with them. In the numeracy condition, participants were told that “children can count, compare, and measure the bears.” In the spatial condition, participants were told that “children can build, stack, and arrange the bears.” In the patterning condition, participants were told that “children can sort, match, and alternate the bears.” Finally, in the fun condition, participants were told that “when children play with these bears, they often have fun!” After reading the description, participants completed a manipulation check that asked them to select the skill that toy was designed to improve (numeracy, spatial, patterning, or fun). As a note, all but 14 participants passed the manipulation check (95% pass rate). Participants who failed were more likely to be in the “fun” condition (*n* = 10) than in the numeracy (*n* = 1), spatial (*n* = 2), or patterning (*n* = 1) conditions, chi-square (3, *N* = 279) = 16.18, *p* = .001. Results were largely unchanged when we removed these 14 participants, so we report analyses on the full sample.

Participants then answered the same questions presented in Study 1. Three primary questions were about the toy − (1) “In general, how likely are parents in the US to buy this toy for their son?”; (2) “In general, how likely are parents in the US to buy this toy for their daughter?”; and (3) “How much would you have liked this toy as a child?” Participants also answered five additional items which we do not report on here. At the end of the survey participants answered key demographic questions and were asked to leave any comments or issues they had about the survey if desired. The full list of survey items is available on the Open Science Framework (OSF) at https://osf.io/zrjm4.

## Results

### Perceptions of Parents’ Willingness to Buy Toy

**Fig. 3:**
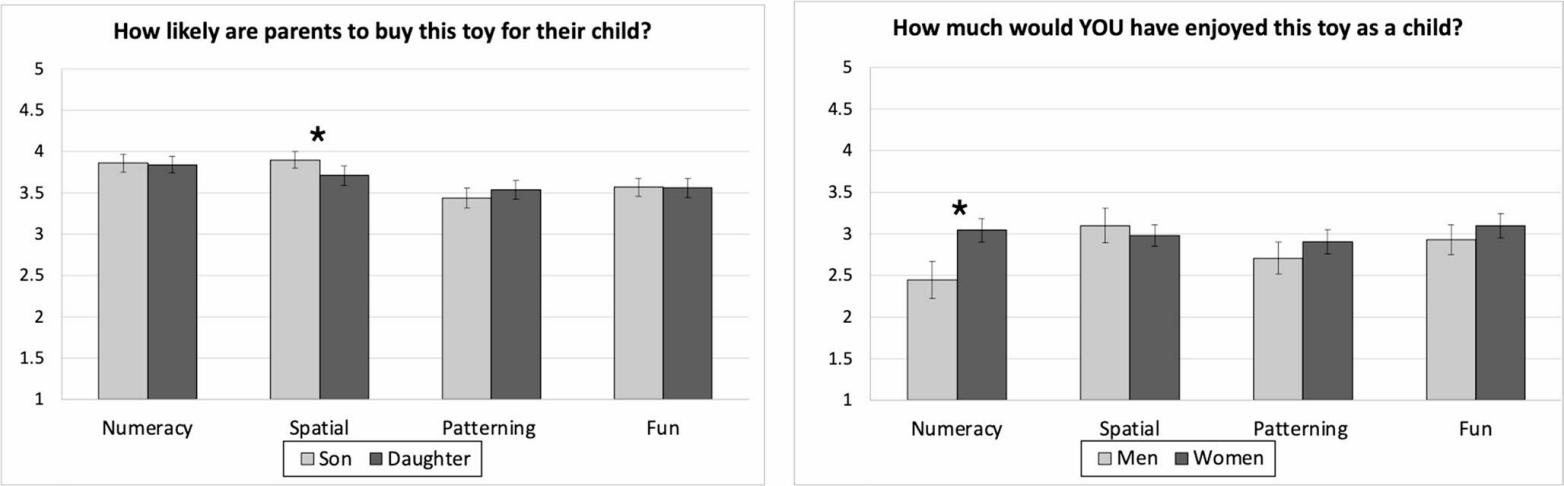
Panel **A** - Perceptions of parents’ (in the US) willingness buy the toy bears for child (boy or girl), as a function of cognitive skill*.* Panel **B** - Ratings of how much the participant would have enjoyed the toy as a child, as a function of cognitive skill. *Note.* Error bars represent ± 1 standard error. * indicates *p *< .05, ** indicates *p *< .001. The sample consisted of *n *= 268 in Panel **B**

To examine differences in ratings across gender and skill framing, we conducted a 2 x 4 mixed ANOVA with target gender (boy, girl) in the item wording, as a within-subjects factor and framing condition (numeracy, spatial, patterning, fun) for the toys presented as a between-subjects factor. See Fig. 3. There was a main effect of the framing condition, *F*(1, 274) = 3.00, *p* = .031, η_p_^2^ = .03. Participants believed parents were more likely to buy spatial (*M* = 3.81, *SD* = 0.94) and numeracy toys (*M* = 3.85, *SD* = 0.86) and less likely to buy patterning (*M* = 3.49, *SD* = 0.98) and fun toys (*M* = 3.55, *SD* = 0.95). Pairwise comparisons indicated significant differences between numeracy and patterning (*p* = .017), between numeracy and fun (*p* = .044), and between spatial and patterning (*p* = .030).

The main effect of target gender, *F*(1, 274) = 0.38, *p* = .536, η_p_^2^ = .01, and the interaction between the two factors were not significant, *F*(1, 274) = 2.03, *p* = .110, η_p_^2^ = .02. Although the interaction did not reach significance, the theoretical importance of the gender differences within spatial task warranted a closer look at the simple effect of the spatial framing. Exploratory post-hoc comparisons revealed significant gender differences in the spatial framing condition, as participants rated that parents were more likely to buy the toy for their sons than for their daughters, *t*(72) = 2.76, *d* = 0.32, *p* = .007. The gender difference was not significant in the other three conditions: numeracy (*d* = 0.03), patterning (*d =* −0.11), and fun (*d* = 0.00).

### Rating of Their Own Toy Enjoyment as Children

We then tested if men and women reported differential imagined toy enjoyment as a child based on the framing condition. We conducted a 2 x 4 ANOVA with participant gender (women, men) and framing condition (numeracy, spatial, patterning, fun) as between-subject factors (see Fig. 3). There was no main effect of participant gender, *F*(1, 267) = 3.00, *p* = .084, η_p_^2^ = .01, no main effect of framing condition, *F*(3, 267) = 1.42, *p* = .237, η_p_^2^ = .016, and no interaction between the two, *F*(3, 267) = 1.35, *p* = .259, η_p_^2^ = .06. While this interaction did not reach significance, results from both Studies 1 and 2 provided evidence to suggest significant gender differences among the rating of toy enjoyment as a child for numeracy and/or patterning, which warranted further investigation. Exploratory post-hoc comparisons revealed a significant gender difference for the numeracy condition, with women rating the numeracy toy as more enjoyable (*M* = 3.05, *SD* = 1.06), compared to men, *M* = 2.44, *SD* = 0.86; *t*(60) = −2.14, *p* = .036, *d* = −0.60.

## Discussion

Study 3 investigated how the math skills labels attached to toy bears influenced adults' perceptions of that toy. The results replicated several key findings from Studies 1 and 2. First, findings are in line with the “spatial as male” stereotype as participants rated that parents would be more likely to buy the toy bears for their sons than daughters when it was framed as a spatial toy. Again however, these gendered preferences were not present when participants reported their own liking of the toy bears framed as supporting spatial reasoning. Second, the results for numeracy and patterning toys were not particularly strong, though there were hints for a more neutral or female-leaning bias. No gender preferences emerged when rating parents’ purchasing behavior, though women reported liking the bears more than men when labeled as numeracy toys. Third, similar to Study 2, the labels mattered. For example, even though the toy image was identical across conditions, participants thought parents would be less likely to buy the toy when it was labeled for patterning and fun.

## General Discussion

Across three studies, we assessed adults’ gendered perceptions of toys that were associated with numeracy, spatial reasoning, and patterning. In Study 1 (within-subject), we selected toys that were associated with these constructs based on their website descriptions, but the participants made judgments based solely on the visible features of the toys. In Study 2 (within-subject), we selected a variety of spatial toys that varied in their features and labelled them as supporting numeracy, spatial reasoning, patterning, or just for fun. In Study 3 (between-subject), we selected one neutral toy, and adults were randomly assigned to view that toy with one of the four labels: numeracy, spatial reasoning, patterning, or just for fun.

Several findings emerged. First, participants consistently associated toys supporting spatial reasoning with boys over girls revealing a “spatial as male” stereotype. In Study 1, participants rated boys as liking spatial reasoning toys more than girls. In Studies 2 and 3, participants reported that parents in the United States would be more likely to purchase toys which support spatial reasoning for their sons over their daughters. Second, the results for numeracy and patterning toys were mixed, but at times there tended to be an attenuated or even reversed perception. In Study 1, women reported more liking than men for both patterning toys and numeracy toys, and this finding was replicated for patterning toys in Study 2 and numeracy toys in Study 3. Third, perceptions varied depending on whether the adults were thinking about others (e.g., parents buying toys) versus themselves as children, and tended to have a stronger traditional leaning toward boys when considering others. Fourth, adults’ perceptions of the toys seemed to be influenced both by the specific toy features and by the labels. For example, in Study 1, adults frequently mentioned the perceptual, physical, and component parts of the toys, suggesting these features mattered. However, in both Study 2 and Study 3, adults thought parents would be least likely to buy a toy if it was “just for fun,” suggesting the labels mattered.

### Adults’ Gendered Perceptions of Skills for Children

Why do adults align spatial toys more with boys than with girls? One possibility is that adults consider spatial toys to be more educational in nature, and they assume parents are more likely to purchase educational toys for their sons than for their daughters. This explanation may play a role, as adults in Study 1 mentioned how the spatial toys promoted learning and creativity. However, we think it is unlikely to fully explain these effects, as it was often the numeracy toys that were rated more highly for supporting academic math skills. Another possibility is that adults perceive the spatial toys we used in these studies as more physical and hands-on relative to the other toys, and they associate this physicality with boys. This explanation seems more plausible as (a) we selected spatial toys that afford object manipulation, and (b) adults in Study 1 very frequently mentioned the physical building and fitting together aspect of the spatial toys. This is consistent with previous research that suggests activities involving building and construction are often considered more typical or prominent for boys than for girls (e.g., Connor & Serbin, 1977; Doyle et al., 2012).

One surprising finding was the lack of evidence to support the hypothesis that numeracy toys would be associated with boys. Participants tended to rate toys which supported numeracy as equally associated with girls and boys. Further, in Studies 1 and 2, women reported they would have liked numeracy toys more as a child than men. One possibility is that the adults perceived “numeracy” as narrower than math as a whole and did not align it with men or boys. This seems unlikely given that the adults in our study did associate the numeracy toys with the term math more broadly. For example, participants rated the numeracy toys as highly likely to support math achievement. Another possibility is that the adults equated numeracy with math in their mind, but because the “math as male” stereotype is so well known, participants may have engaged in social desirability and not reported a difference in their perception of these toys for girls versus boys. Another explanation is that the stereotype of “math as male” is diminishing over time, as gender parity is now often occurring in the biological sciences and mathematical fields (Eagly, 2021). The lack of evidence for a strong “numeracy as male” stereotype could be a strong leverage point for considering how to approach math without male-stereotypic associations, but future research is needed to more carefully disentangle numeracy from the broader mathematics stereotypes.

The results also showed mixed findings for toys associated with patterning. There was not strong or consistent alignment with either boys or girls, but in both Studies 1 and 2 women rated their enjoyment higher for patterning toys than males. Further, Study 1, there were ratings of girls liking three of the four patterning toys more than boys. Perhaps this leaning toward “patterning as female” is because many common patterning tasks involve arts and crafts that are associated with girls and women. For example, common patterning tasks in childhood include painting alternating stripes for art or creating a pattern with beads on a string (e.g., Ginsburg et al., 2003). These results suggest that at least some components of mathematics do not hold strong gendered associations and could have implications for considering the role of girls and women in STEM fields.

### Adults Preference of Skills for Self

These studies show more evidence of gender stereotypic preferences in beliefs about *others’* behavior than in beliefs about *one’s own* past attitudes. This pattern is generally consistent with other findings of more stereotypic expectations applied to others than the self. For example, participants report cultural beliefs about prejudice to be stronger than their own personal beliefs (Devine, 1989). More broadly, this pattern echoes past work finding that people expect others to be more susceptible to cognitive and motivational biases, such as the fundamental attribution error or halo effect (Pronin et al., 2002). This reduction in stereotypicality potentially supports the notion that girls can and do enjoy mathematical tasks.

This finding might align with the hypothesis that girls and boys are exposed to different degrees of spatial task interactions not because of differences in their own interest but because of caregivers' perceptions of task appropriateness. For example, in one study, the quality of parent-child puzzle play was higher for boys than for girls, meaning that parents were more engaged with their sons during the task than they were with their daughters (Levine et al., 2012). In another study, parents and three-year-old children interacted with a spatial task app and parents talked more about shape with their sons than their daughters (Verdine et al., 2019). Finally, in dyadic mother-child engineering tasks, mothers spent more time building with sons but reading with their daughters (Coyle & Liben, 2020). This evidence further suggests the importance of disentangling adults’ perceptions of children’s preferences from children’s actual preferences to fully understand the origin of gendered perceptions of certain skills and the downstream performance impacts.

### Limitations and Future Directions

Several limitations of the current investigation suggest directions for future research. The first set of limitations are methodological concerns. The participants in Studies 1 and 2 were convenience samples, based on college students. Further, while Study 3 achieved a more representative sample in terms of age and experience, all study samples were disproportionately female. Another limitation of these studies is that it is not always clear why participants reported certain perceptions and preferences for the toys. In Studies 2 and 3 we attempted to focus participants' attention directly on the cognitive skills (as opposed to just the visible toy features) by labeling the toys; however, we did not ask participants directly which features influenced their ratings, thus it remains unclear exactly which features or other underlying factors influenced their perceptions and preferences. Future research should move beyond Likert scale self-reports and use participants' explanations to determine the extent to which they are spontaneously highlighting certain features of the toys, or mentioning demographics such as gender to describe who would like these toys and why.

The most significant limitation of this study is that it was based on self-report preferences. It is unclear whether participants’ gendered perceptions of these skills would influence what toys they would purchase or how they would interact with these toys with children in real world contexts. Future research should continue to understand the behavioral implications of adults' perceptions on children’s learning, as parents are particularly influential in children’s early math learning (Daucourt et al., 2021; Hornburg et al., 2021). By analyzing parents' behaviors with children and tasks perceived in different ways, we can better understand the extent to which these gendered perceptions of tasks matter for children’s learning.

### Practice Implications

Our results have at least two potential applications in practice. The first is related to toy selection. Our findings suggest that boys and girls may have more varied preferences than typically assumed (based on the adults’ ratings of their own liking of the toys in our three studies), and that we should offer a variety of toy types to boys and girls. Also, our results show that patterning and numeracy toys hold weaker gender stereotypes compared to spatial toys, suggesting that promotion of these activities by parents and educational practitioners may be less likely to prevent the activation of certain biases and could potentially promote increased gender-equity in the engagement of math tasks.

The second is related to labels and descriptions applied to learning toys. Our results suggest that the exact same toy, just with a different label, can be perceived as more desirable for all children across the board or for a specific kind of child (e.g., boys), making it more likely to be purchased by caregivers. Marketers and educators may need to think critically about the ways in which they frame early childhood activities to avoid gendered associations. This is consistent with evidence that even subtle framing of tasks in educational contexts can influence children’s behaviors. For example, a study with elementary school children revealed underperformance effects in a working memory task when it was framed as a math task but not when it was framed as a spatial task (Hildebrand & Cordes, 2024).

## Conclusion

While the “math as male” stereotype is well established, especially within Western countries, mathematics knowledge requires several related cognitive skills. This investigation teased apart the gendered associations of three of these cognitive skills: numeracy, spatial reasoning, and patterning. The results suggest a “spatial as male” stereotype when considering the types of toys associated with boys. Further, they suggest that this gender stereotype is attenuated for toys associated with patterning and numeracy. Finally, adults’ perceptions differed when considering others versus the self, with a shift away from the “spatial as male” stereotypes. Understanding how adults associate these cognitive skills with gender has important implications for research in mathematics education and belonging within STEM fields more broadly. More research is needed to uncover the extent to which even subtle framing of tasks in educational contexts can influence parents, teachers, and children’s engagement with STEM activities.

## Figures and Tables

**Figure:**
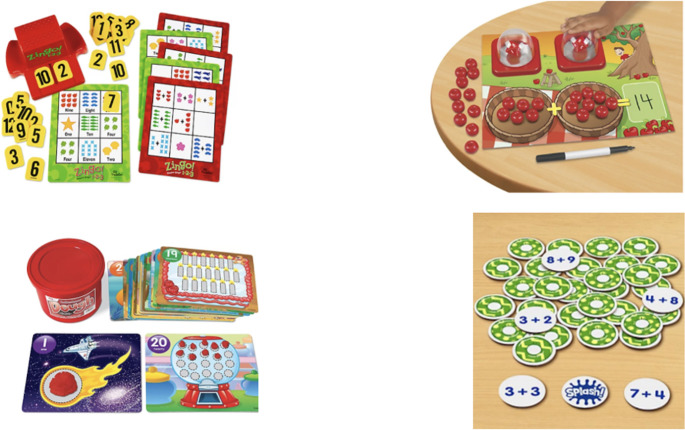
Numeracy toys

**Figure:**
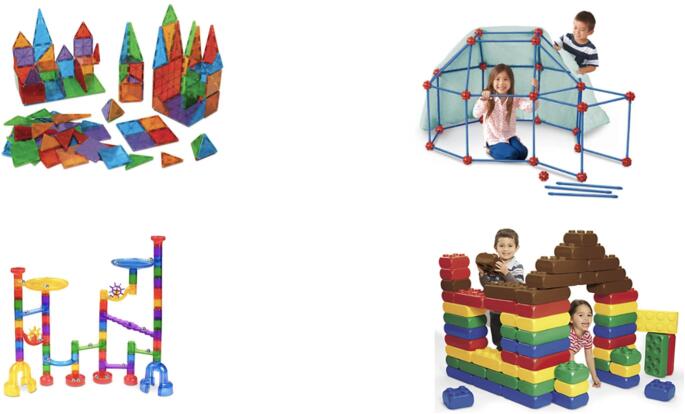
Spatial toys

**Figure:**
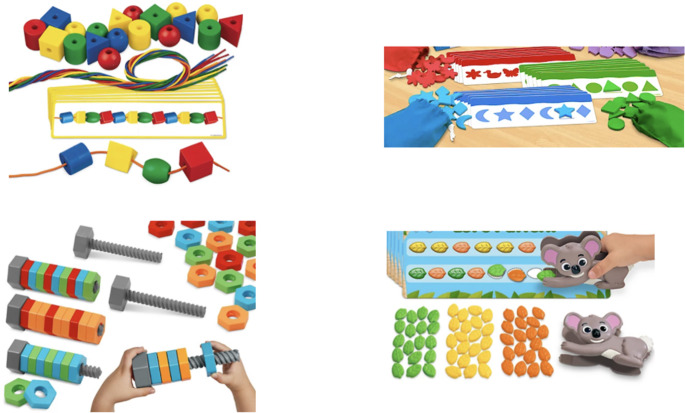
Patterning toys

**Figure:**
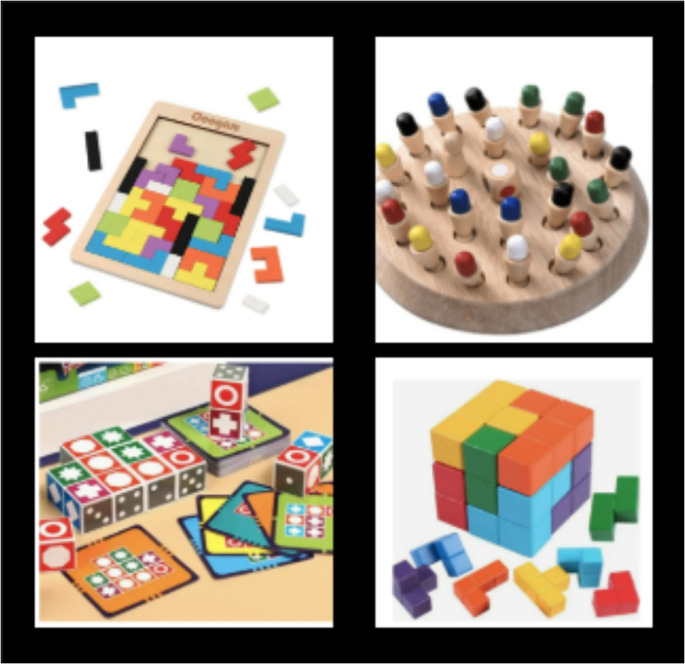
Stimuli for study 2

**Figure:**
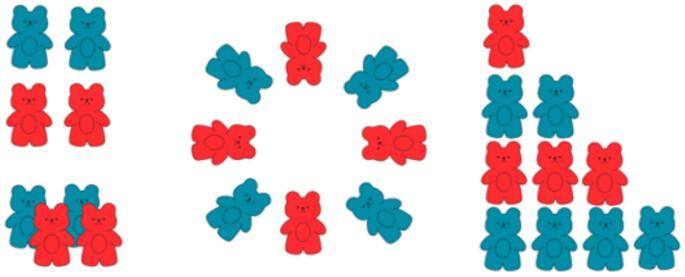
Stimuli for study 3

## Data Availability

Data from all three studies are available on the Open Science Framework (OSF) at https://osf.io/zrjm4.
